# Combined Effects of Social Exclusion and Social Rank Feedback on Risky Decision-Making Across Adolescence

**DOI:** 10.1007/s10964-024-02072-w

**Published:** 2024-08-28

**Authors:** Corinna Lorenz, Nicola K. Ferdinand

**Affiliations:** Department of Psychology, University of Wuppertal, Wuppertal, Germany

**Keywords:** Adolescence, Risky decision-making, Social exclusion, Peer influence, Social rank, Reward processing

## Abstract

Adolescents’ need to belong and concerns about social status are thought to increase risk-taking, however, not much is known about how feedback about social rank and the effects of social exclusion moderate risky decision-making. To this end, the present study examined how social rank feedback moderates the effects of social exclusion on risky decisions during adolescence. The experimental study included a total of 122 participants (11–19 years; 44% female). Participants were randomly assigned to receive either individual or social rank feedback in the Columbia Card Task after social inclusion and exclusion via the Cyberball paradigm. Contrary to expectations, social exclusion led to more cautious decision-making. Mid-adolescents were most influenced by the combination of social exclusion and social rank feedback, while late adolescents became more cautious with individual feedback. These findings suggest that peer influences also have adaptive effects, increasing sensitivity to risk information, with developmental differences in the role of social rank.

## Introduction

One of the primary developmental goals during adolescence is the achievement of independence, which facilitates the formation of new and more mature interpersonal relationships with peers (Havighurst, 1953). Increased time spent with peers (Brown & Larson, 2009) and an increased need to belong (Tomova et al., 2021) correlate with the acquisition of social skills, but also with delinquent behavior and risk-taking (Weerman et al., 2015). This risk-taking may stem from a strong drive for acceptance, where fear of social exclusion outweighs concerns about negative outcomes (Tomova et al., 2021). The social reorientation from family to peers causes adolescents to become preoccupied with belonging and therefore to begin to behave in accordance with social goals, such as achieving higher social status or rank (Op de Macks et al., 2017). At the same time, peer groups are in a constant state of flux (Hitti et al., 2011), and adolescents are most likely to engage in and experience acts of social exclusion (Mulvey et al., 2017), while also showing more negative affect (Sebastian et al., 2010) and likely long-term consequences than other age groups (Mulvey et al., 2017). Thus, understanding the interplay between decision making and social pressures such as social exclusion and social rank is critical to mitigating negative peer influences and fostering positive peer relationships as adolescents move toward independence. In this study, social exclusion and inclusion were experimentally manipulated to examine how being excluded from a virtual peer group influences risky decisions and information use, assessed through a task evaluating sensitivity to gain amounts, loss amounts, and loss probabilities, while also exploring the moderating effects of feedback on social rank.

### Peer Influence on Adolescent Risk-Taking

The dynamics of social influence and its impact on risky decision-making among adolescents is a complex area of research. In the laboratory, peer influences have been studied using a variety of methods, with peers merely present, observing, or actively encouraging risk-taking behavior (for review, see Powers et al., 2022). Some models suggest that peer influence triggers impulsive decision-making due to an imbalance between an immature cognitive control system and a hyperactive system for processing socioemotional information (Shulman et al., 2016). Overall, experimental evidence supports the notion that the presence of peers increases risk-taking tendencies during adolescence. Neurodevelopmental approaches have shown that adolescents are more risk-taking in the presence of peers and show more activity in reward-related brain regions, such as striatal areas, in such situations than any other age group (Chein et al., 2011). As such, peer presence is thought to be a rewarding situation, causing impulsive decisions, increasing risky choices and the perceived value of potential rewards as opposed to thinking about consequences (Shulman et al., 2016).

Alternative perspectives suggest that peer influence can foster trust and social learning. Adolescents may engage in goal-directed behavior and calculated risk-taking to gain social status and acceptance within peer groups (Ciranka et al. 2021a). Accordingly, meta-analytic evidence suggests that the effect of mere peer presence is small but robust, with high variance in contextual settings, and becomes strong only when peers actively encourage risk-taking or express pro-risk attitudes (Powers et al., 2022). It has been emphasized that adherence to peer norms may be enhanced by an ongoing need to belong to peer groups. Specifically, it has been proposed that the pervasive fear of social exclusion during adolescence may contribute to increased risk-taking as a means of regaining acceptance and restore social status (Tomova et al., 2021).

### Social Exclusion and Social Rank

Social exclusion has been studied in the laboratory using virtual environments in which participants are ignored or excluded from activities. One such environment is the Cyberball paradigm (Williams & Jarvis, 2006), in which participants are tasked with a simple ball-tossing game. While engaged in the task of mentally representing the setting, environment, and people, they play the ball-tossing game with virtual others. In the case of social exclusion, the confederates stop tossing the ball to the participant, creating a feeling of being left out of the group activity. The effect of social exclusion has been shown to be robust against distrust in the realness of the environment, with long-lasting effects on mood and behavior (Hartgerink et al., 2015). In terms of effects on adolescent risk behavior, it has been shown that thinking about others during social exclusion is associated with risky driving in the presence of peers (16–17 years, Falk et al., 2014). Similarly, adolescents (15–17 years) with low peer resistance show higher levels of risky driving following social exclusion (Peake et al., 2013). In adults, social exclusion sometimes showed no effects on risky decision-making (Murphy, 2019), or increased risk-taking (Meng, 2020; Twenge et al., 2002), sometimes also only when an opportunity was given to regain acceptance through such behavior (DeWall & Richman, 2011; Mead et al., 2011).

Accordingly, in addition to the risk-increasing effect of social exclusion on adolescents, experimental studies support the importance of social standing in adolescent risk-taking. Social standing, or rank, refers to one’s position relative to others within a social hierarchy, which can be derived from, for example, subjective popularity or task performance (Koski et al., 2015). Occupation with topics such as social rank thereby peaks during adolescence (LaFontana & Cillessen, 2010). Accordingly, in a task where adolescent girls could bid on items, they often overbid to win bets that were revealed to fictive peers, meaning that they tolerated high financial losses to show that they were winning over others (Cardoos et al., 2017). However, compared to monetary feedback, i.e., winning or losing more or less money, increasing or decreasing social rank relative to others did not affect risk-taking in adolescent girls (Op de Macks et al., 2017). It is possible that persuasive peer interactions and the knowledge that others will notice changes in social rank are crucial for inducing effects on risk-taking. Moreover, by making risk-taking behavior relevant to the social situation at hand and thus goal-directed behavior, integrating social rank into the study of peer effects on adolescent risk-taking may enhance these effects.

### Developmental Differences in the Use of Information

Risk-taking, status-seeking, and fear of social exclusion are collectively attributed to neurodevelopmental changes and developmental tasks during adolescence, these phenomena have rarely been studied simultaneously to capture their interrelationships. In addition, it remains to be determined whether this socioemotional vigilance is actually maladaptive or whether concern for peer social status may also be an adaptive feature of adolescence. Many dynamic experimental risk-taking tasks provide valuable insights into how adolescents explore uncertain but risky situations, enhancing ecological validity in contrast to static decisions under known risks. Accordingly, dynamic risk-taking has been associated with real-life risk behaviors, such as drinking (Weber & Johnson, 2009). However, they often fail to fully capture adaptive behaviors, such as the processing and incorporation of risk information. In contrast, the Columbia Card Task (CCT, Figner et al., 2009) assesses decision-making under risk by having participants select a series of cards that represent either small but recurring gains or large and infrequent losses. This allows researchers to study how individuals adapt their risk-taking to different aspects of the situation, such as varying amounts of gain, loss, and probability of loss, which are fully provided for each series of choices. As such, the CCT provides a promising task environment for investigating how the presence of peers affects information use in decisions that are still affectively arousing (Schonberg et al., 2011).

Developmental models suggest a maturational imbalance between socioemotional arousal and cognitive control (Shulman et al., 2016). Accordingly, the reward sensitivity associated with this maturational imbalance has been shown to peak in mid-adolescence in developmental studies on risky decision-making (Braams et al., 2016; Chein et al., 2011) but also cognitive control (Ferdinand et al., 2022) and other task settings (Kray et al., 2018). Studies using the CCT have shown how adolescents’ decision-making tendencies are consistent with these developmental trends. Findings revealed that mid-adolescents between the ages of 14 and 16 incorporate less information about risk and reward than adults, which may contribute to their increased risk taking (Figner et al., 2009). However, another study reported no significant age differences in sensitivity to information about gain and loss amounts and probabilities across a broader age range (8–35 years, Duijvenvoorde et al., 2015). Investigations of social influences on CCT suggest that the presence of peers may affect decision making in different ways depending on task context and age group. Specifically, findings suggested that the presence of peers actually increased risk-taking in early to mid-adolescents (ages 13–15 and 16–18), but older age groups (ages 19+) responded in the opposite way, making less risky decisions in the presence of peers than alone (Somerville et al., 2019). As such, information use is a sensitive measure to reflect developmental differences in social influences on risky decision-making across adolescence.

## Current Study

Despite significant advances in understanding social influences on adolescent behavior, several gaps remain. First, while social cognition significantly influences adolescents’ decision making, the effects of social exclusion and its association with social status are underexplored. Most research has focused on simulated driving tasks, neglecting how social exclusion might affect the processing of potential gains and losses and their probabilities. Furthermore, the effects of social exclusion on risky decision-making have not been compared across developmental stages, despite peaks in reward sensitivity and social vulnerability during mid-adolescence. For these reasons, this study examined the combined effects of social exclusion and social rank feedback on adolescent risk-taking decisions. It was expected that socially excluded adolescents would exhibit increased risky decision-making compared to socially included adolescents (Hypothesis 1a). More specifically, it was hypothesized that increased risky decision-making following social exclusion would result in decreased use of information about gains, losses, and loss probabilities (Hypothesis 1b). Given adolescents’ sensitivity to social rank, it was also predicted that under combined conditions of social exclusion and social rank feedback, adolescents would make riskier decisions (Hypothesis 2a) due to reduced information use (Hypothesis 2b). Finally, it was hypothesized that while adolescents generally improve in their use of information about gains, losses, and loss probabilities with age (Hypothesis 3a), mid-adolescents would show the greatest effects of combined social exclusion and social rank feedback (Hypothesis 3b).

## Methods

Please note, this study’s desired sample size, included variables, hypotheses, and planned analyses were preregistered on Open Science Framework (https://osf.io/aywvb/).

### Participants

The final sample for analysis consisted of *N* = 122 participants that were randomly assigned to one of the two feedback groups. 58 individuals (39.66% female) received individual feedback, which consisted of information solely about their accrued points, and 64 individuals (48.44% female) received feedback in the form of a social rank, revealing their points earned in comparison to two virtual peers. Please note, sex was recorded by asking for the sex as listed on the birth certificate, allowing for any changes and entries outside the binary sex system to be specified. All participants identified their sex as either female or male. The mean age of the individual feedback group was 15.3 (*SD* = 2.7, age range = 11.0–20.1 years) and for the social rank feedback group it was 15.4 (*SD* = 2.5, age range = 11.1–19.8 years). Among the participants, 78.69% reported German as their mother language, while 4.10% reported Arabic. All other mother tongues each comprised less than 4% of the total sample. Of the total sample 81.15% of the participants still went to school with 76.77% visiting schools preparing for academic certificates. One participant reported to be still in Primary school. Participants that reported to have left school had all acquired a qualification that grants access to universities.

Adolescent participants were recruited through a multi-faceted approach in the vicinity of the University of Wuppertal in Germany in the years from 2021 to 2023. Recruitment methods included the distribution of flyers in local schools, organizations, and at public events. Late adolescents were also recruited from the pool of undergraduate university students utilizing the SONA Participant Recruitment System. Potential participants were informed that they could participate in the study if they met the following criteria: fluency in the German language and the absence of a history of psychological, neurological, or learning problems. To acknowledge their participation, eligible participants were compensated with a 10€ gift card or received participation credits if they were enrolled in psychology, sociology, or sports science. From 124 participants that were enrolled in the study, two participants from the individual feedback group were excluded prior to analysis as they showed extreme values and a notable lack of decision variance in the decision-making task. More specifically, one participant consistently selected either none or all of the cards in the trials of the CCT and the other one most often chose all cards. Two participants were excluded from the manipulation check analyses of the questionnaire data because the participants did not complete the questionnaire due to technical problems.

### Procedure

The experiment was conducted using computer-based setups, which included a laboratory setting as well as tablets deployed in schools. Participants underwent testing individually or in groups, with group sizes limited to a maximum of three individuals. To ensure that there were no social interactions between the participants, despite being tested in the same room, the participants were not allowed to communicate with each other. They were placed in individual workspaces and informed that they should not interact. In addition, participants were informed throughout the procedure that the other players with whom they were interacting were in a different room or lab.

**Fig. 1:**
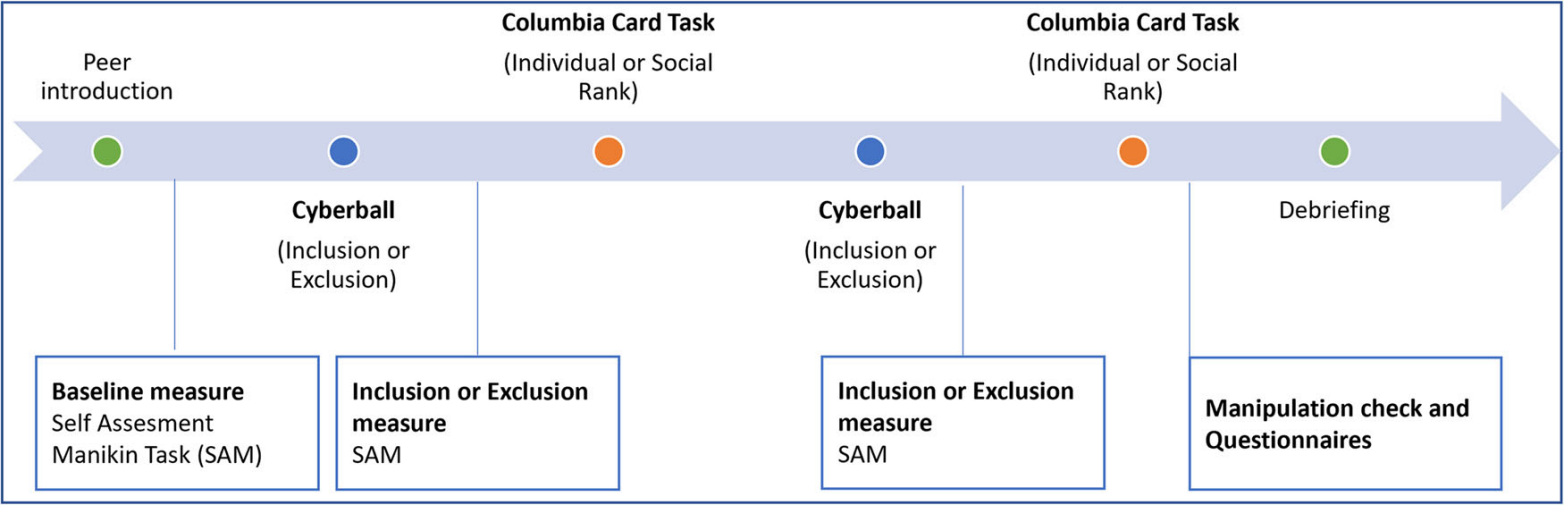
A graphical representation of the procedure. Participants were randomly assigned to one of two feedback groups in the CCT, as well as to one of the two groups either receiving the inclusion or exclusion condition in the Cyberball paradigm first

The following section will present the material in the order of their occurrence in the paradigm. The experimental procedure is graphically illustrated in Fig. 1. First, participants were introduced to two virtual peers via a simulated chat environment. In the Cyberball paradigm, participants were either included or excluded from the group by receiving the ball during a game, either for the same amount of time or only at the beginning. Following each round of the Cyberball game, participants engaged in a risky decision-making task, the Columbia Card Task (CCT), and received either individual feedback or feedback in the form of a social rank. Following the introduction of the peers and each iteration of the Cyberball game, participants were prompted to indicate their emotional state via the Self-Assessment Manikin Task (SAM). This was followed by a series of questionnaires designed to measure further information regarding their cognitive and emotional responses during the tasks, following the conclusion of the experimental procedure.

Upon their arrival at the testing location, participants underwent random assignment. To ensure a balanced gender ratio across feedback groups and to maintain the integrity of random assignment, participants were first stratified by three age groups (11–13, 14–16, and 17–19 years) and gender. Each participant was then assigned a unique ID code to ensure an equal number of male and female participants in each age group. Within these strata, participants were then randomly assigned to either the individual feedback group or the social rank feedback group. Additionally, the order of experiencing social exclusion (first or second) was randomized. In addition to the questionnaires used to examine the effects of social exclusion as described in the following section, also resistance to peer influence, rejection sensitivity, and need to belong were measured. Adding additional research questions regarding individual differences to the ones already included in this manuscript would go beyond the scope of this study. Thus, individual differences in how social exclusion and social rank affect risky decisions in adolescence will be reported elsewhere.

### Material

#### Peer introduction and cyberball paradigm

**Fig. 2:**
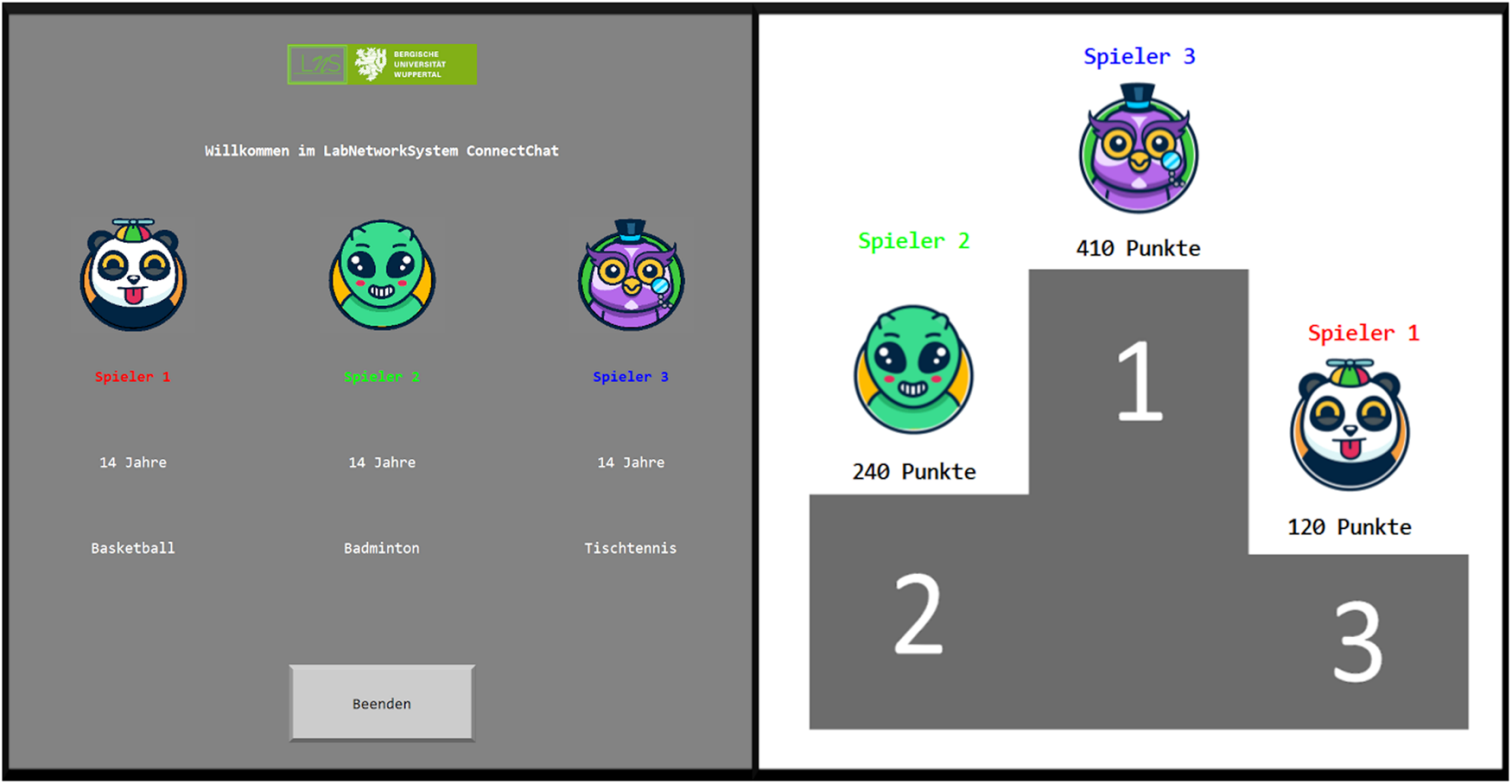
**Left:** To acquaint participants with their fictitious confederates, participants generated a personal profile as Player 2, which included their age, a chosen hobby, and the selection of an avatar to represent themselves throughout the duration of the experiment. **Right:** Following each of the two rounds of the Columbia Card Task (CCT), participants in the social rank group were provided with feedback regarding their performance compared to the fictitious confederates

**Fig. 3:**
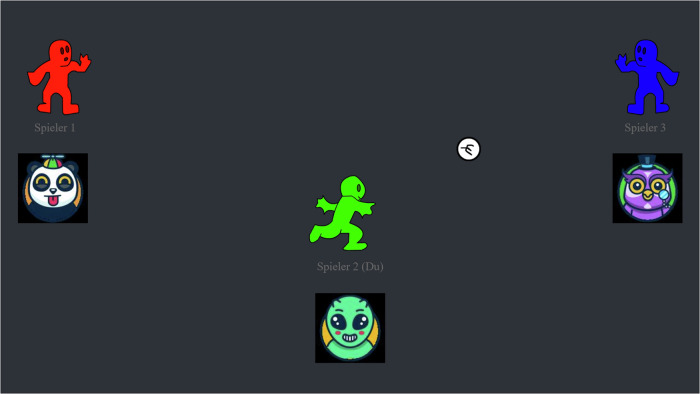
In the Cyberball paradigm, participants were instructed to enhance their mental visualization skills by engaging in a virtual ball tossing game, all while visualizing the game’s surroundings and other players. During the social inclusion condition, all players received the ball with equal frequency. In contrast, during the social exclusion condition, participants were allocated the ball only at the commencement of the game and once during its progression, creating an experience of being left out by the other players

To enhance the authenticity of the social interaction, participants were introduced to two virtual peers through a simulated university network. The virtual peers were supposedly connected to the network sitting in other labs. The participant created profiles that included a selected avatar, a disclosed hobby, and their age. Subsequently, the own profile and the two other preformulated profiles belonging to the virtual peers were presented to the participants, creating a semblance of genuine social engagement (see Fig. 2). Following the introduction to the virtual peers, the participants’ perceptions of inclusion or exclusion within a social group were manipulated using the Cyberball paradigm (Williams & Jarvis, 2006). The aim was to investigate the impact of social exclusion on risky decision-making. The participants were directed to train their mental visualization skills by immersing themselves in the ball tossing game. To this end, participants were asked to visualize the game’s environment and interact with the two fictitious peers they had previously encountered virtually (see Fig. 3). In the inclusion condition, participants received the ball at a frequency equivalent to that of the other players, which was approximately one-third of the total ball tosses. Conversely, in the exclusion condition, participants were deliberately assigned the ball in only 16% of the ball tosses (5 out of 30 tosses). To circumvent any potential bias that might arise from the sequence in which the inclusion and exclusion conditions were experienced, a counterbalancing approach was employed across participants.

#### Risky decision-making

**Fig. 4:**
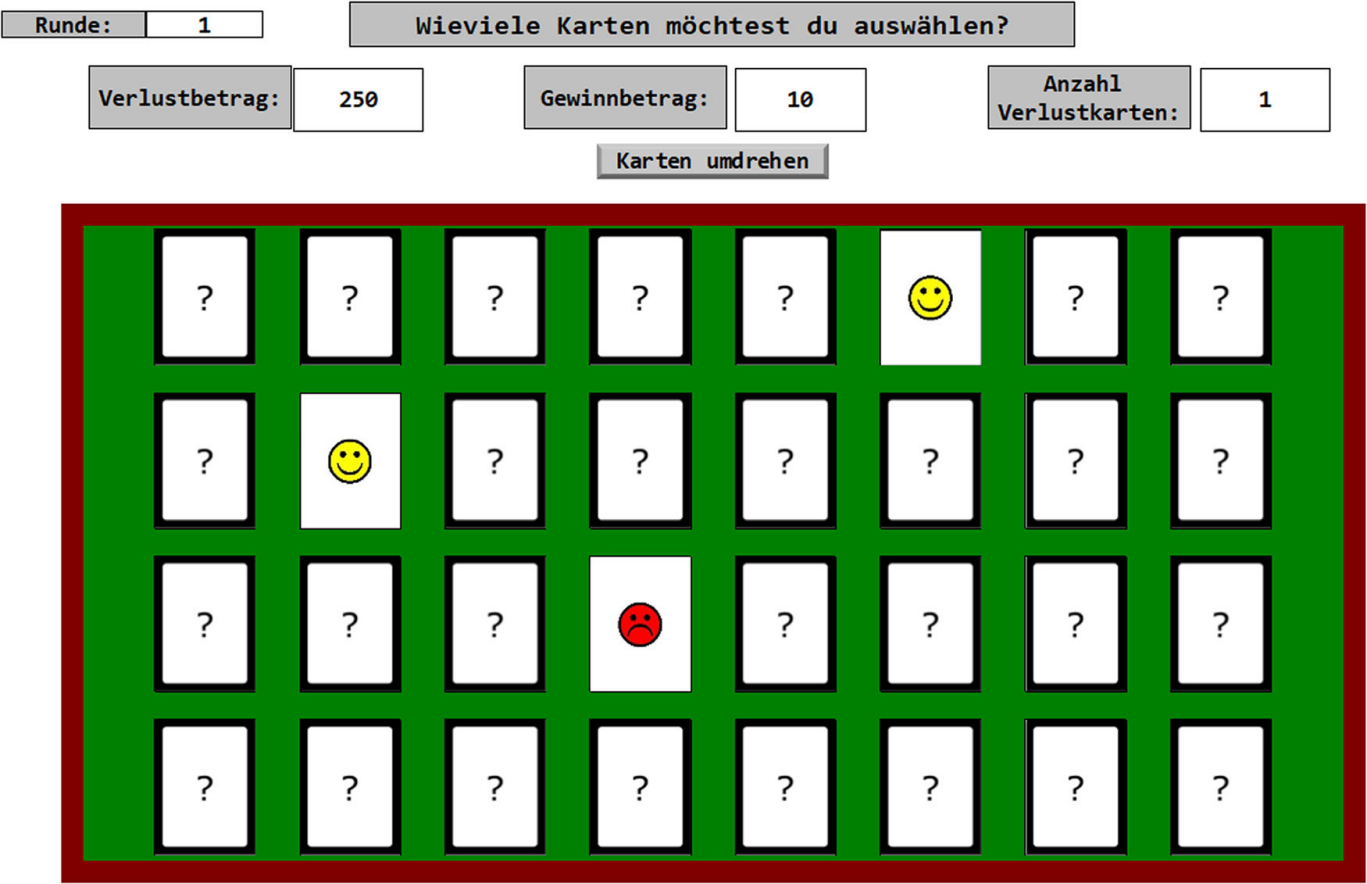
In the Columbia Card Task, participants make selections to reveal specific cards from a set of 32 face-down cards. Each card in this array carries a distinct probability of either yielding a gain or a loss. Trial information (“Runde”), including gain amounts (“Gewinnbetrag”), loss amounts (“Verlustbetrag”), and the number of loss cards on the table (“Anzahl Verlustkarten”), is prominently displayed at the top of the screen for every trial

To assess risky decision-making, a modified version of the Columbia Card Task (CCT, Figner et al., 2009) was employed in E-Prime 3.0 software (Psychology Software Tools, Inc. 2016) based on Murphy (2019). In this computerized task, participants are presented with a table containing 32 face-down cards organized into four rows. These cards display either smileys (gain cards) or frownies (loss cards) on their face sides (see Fig. 4). At the upper section of the screen, information for each trial was displayed, including the gain value for positive cards, the loss value for negative cards, and the number of loss cards that could be encountered in the deck. Gain cards were assigned point values of either 10 or 30 points, while loss cards incurred penalties of either 250 or 750 points. The deck contained either 1 or 3 loss cards at any given time. In a completely factorial design, every combination of these factors was presented twice, resulting in a total of 24 trials. Each participant underwent two iterations of the CCT, one following their inclusion and one following their exclusion from the cyber ball game. The order of occurrence for both iterations was randomized. Risky decision-making was measured by the number of cards chosen in each trial. A higher number of cards selected signified a greater inclination toward risk-taking, as the probability of encountering a loss card increases with each additional card turned over.

For each trial, participants were tasked with choosing which cards they would like to reveal by marking the face-down cards and confirming their choice with a “turn over” button. It is important to note that unlike the “hot” version of the CCT, this adapted task does not employ risk censoring. This means that through the separation in selection and feedback phase, trials do not terminate upon the choice of a loss card, with the number of cards chosen indicating the non-censored willingness to engage in the risk of losing points (Buelow & Blaine, 2015; De Groot & Thurik, 2018). In a subsequent feedback phase, the selected cards were turned over one by one in order of selection, revealing either a gain or a loss. Which card led to losses was randomly chosen at the beginning of the trial., i.e., the task feedback was not rigged and allowed to experience real outcome probability. For each trial, points linked to gain cards were added to a virtual balance that started at zero points at the beginning of each CCT round and then the next card was turned over. On the other hand, when the first loss card was revealed, its associated points were subtracted and no further cards were revealed. Feedback presentation ended after the first negative card to make outcomes comparable to the original version of the CCT referred to as “hot” (Figner et al., 2009), i.e., it was avoided that multiple losses in a trial occurred as it would fundamentally change scores and probability calculation in the task.

Furthermore, the study investigated whether the sensation of being under evaluation by peers would modulate the effect of social exclusion on risky decision-making. Therefore, one group received individual feedback about their performance, while another group received feedback in the form of a social rank also featuring the confederates’ avatars and points at the end of the two rounds (see Fig. 2), respectively. In both rounds, the social rank feedback consistently depicted participants as the second-highest performer among the group of three players. In order to achieve this, the scores of the virtual peers were calculated by subtracting either 120 or 150 points from the participant’s score for Player 1, and adding 170 or 140 points to the participant’s score for Player 3, respectively, in the first and second round of the CCT. Before engaging in the task, participants were explicitly informed whether the points earned by players would be openly disclosed (in the form of social rank feedback) or kept confidential (provided as individual feedback).

#### Self Assesment Manikin Task (SAM)

To assess emotional responses to the social manipulations used in this study, the Self-Assessment Manikin (SAM, Bradley & Lang, 1994) was employed at multiple time points. SAM assessments were conducted at baseline, following the introduction of fictive peers, and subsequent to each of the two rounds within the Cyberball paradigm. This scale employs a 9-point single-item format to measure three distinct emotional dimensions: arousal, valence, and control/dominance. For each dimension, participants were presented with manikin illustrations representing these emotional scales, encompassing five different images each. The nine points to indicate their emotional state were located at each of the five images and in between them. Although the SAM is a nonverbal measure of emotion, participants were verbally provided with pre-defined adjectives (e.g., happy-unhappy, excited-calm, dominant-controlled) to describe the images of the poles of the scales if participants were unsure of their meaning.

#### Need Threat Questionnaire (NTQ)

To further assess changes in emotions and cognitions during the two Cyberball sessions, a German adaptation (Grzyb, 2005) of the Need Threat Questionnaire (NTQ) (Williams et al., 2000) was used after the experimental session. The NTQ asked about feelings of belonging and selfworth, amongst others, alongside an aversive impact index designed to gauge shifts in feelings subsequent to inclusion or exclusion from the peer group. Items that asked about general emotions were summed to a “Mood” index with higher values signifying a better mood and questions that inquired about a sense of belonging to a “Belonging” index with higher values indicating a stronger feeling of belonging to the peer group. Furthermore, one component of the NTQ inquired about the participants’ subjective estimation of the percentage of balls they received during the Cyberball paradigm, which was designed to assess whether they actively sensed social exclusion.

### Statistical Analyses

#### Statistical power

A power analysis from a pilot study indicated that a minimum sample size of *N* = 30 per feedback group (individual/social rank feedback) is necessary to reliably (*α* = 0.90) detect medium (${\eta }_{\text{p}}^{2}$ ~0.1) and small (${\eta }_{\text{p}}^{2}$ ~0.01) effect sizes for the primary interaction of Feedback Type * Cyberball Condition in a between-within subjects design. Although the pilot did not include age differences, effect sizes were expected to be higher in an adolescent sample, increasing the power to detect effects of social manipulations in this as opposed to the pilot study. The study also examined higher-order interactions, including three- and four-way interactions with information about risk. This information is repeated in the CCT in a factorial manner over 48 trials per participant, allowing for a strong measurement of sensitivity to changes in gain amount, loss amount, and loss probability. While the sample size was not calculated for these complex effects, they were pre-registered and theoretically justified as integral parts of the present research design, viewed as being essential for a comprehensive analysis of decision behavior in the CCT. Consequently, participants were recruited with an a priori target of 30 per age group (11–13, 14–16, 17–19 years) but 122 were ultimately included in the analysis.

#### Linear mixed effect models

For all linear mixed effect models (LMEM), the *R-Studio* (R Core Team, 2023) environment was used. Specifically, the *lmerTest* (Kuznetsova et al., 2017) package was used that is based on the *lme4* (Bates et al., 2015) package but additionally calculates *p*-values based on Satterthwaite’s method for approximating degrees of freedom for the t and F tests. To account for random effects, a maximal random structure with all within-subjects variables and their interactions entering the models as random factors per participant was intended. In the case of convergence issues, the models were reduced to parsimonious ones as recommended by Bates et al. (2015). To this end, first different convergence routines and heightened iterations were applied. If no solutions fitted the model, interactions between random variables were removed from the random structure first. If convergence was still not obtained then random effects with lowest variance explanation were excluded one by one until a model converged. Specific information about convergence of the models can be found in the Appendix.

To evaluate the effectiveness of social exclusion, a manipulation check utilizing LMEM was conducted. These models were employed to scrutinize emotional responses both during the experiment, as assessed by the SAM, and in the post-experiment phase, evaluated through the NTQ. To test hypotheses concerning risky decision-making, a LMEM on the number of cards turned over per trial was conducted. To test the main interaction of interest, i.e., whether social exclusion has combined effects with the social relevance of the risk situation, Cyberball Condition (Inclusion/Exclusion; within-subjects), Feedback Group (Individual/Social Rank; between-subjects), and their interaction entered the model as deviation coded factors (−1/1). Also, the task variables Gain Amount (10/30; within-subjects), Loss Amount (90/270; within-subjects), and Loss Cards (1/3; within-subjects) were included as deviation coded factors (−1/1) and their interactions in the models. To assess potential developmental differences, continuous age was recorded as age in years and months, expressed in fractional years. The mean-centered age variable was entered into the model with linear and quadratic age contrasts to test for potential peaks of the effects in mid-adolescence.

To facilitate visualization and interpretation, breaks in the age variable were marked at the midpoint of three age groups: early (11–13 years; *M* = 12 years), middle (14–16 years; *M* = 15 years), and late adolescence (17–19 years; *M* = 18 years). Although it is common practice to distinguish between different phases of adolescence, it should be noted that the denotation of the three age groups was arbitrary. Simple effects and contrasts were tested to facilitate interpretation of interactions in the LMEM. Given that these follow-up analyses are not independent confirmatory tests but are aimed at understanding the interaction, corrections for multiple comparisons were not applied to avoid increasing the risk of Type II errors (see e.g., Bender & Lange, 2001).

## Results

### Manipulation Check

Overall, the manipulation checks suggested that the manipulation of feeling socially excluded was successful. For more detailed information on the analyses and results of the manipulation check, see Appendix A. The pattern of results obtained by comparing SAM scores at baseline with scores after social inclusion and exclusion suggests that social exclusion significantly decreased positive affect in all groups. Except for mid-adolescents in the individual feedback group, social exclusion had no effect on arousal ratings. Feelings of control were decreased by social exclusion only in late adolescents. Results also indicated that feelings of belonging and mood decreased significantly after social exclusion. In addition, adolescents recognized exclusion from the ball toss game by reporting a lower percentage of balls received in the exclusion condition than in the inclusion condition.

### Risky Decision-Making

To maintain an overview of the effects of the LMEM on the influence of Cyberball Condition, Feedback Group, Age and the information on gain amounts, loss amounts and loss probability, the effects are reported along the announced research questions and hypotheses. The full results of the model can be found in Table 4 in the Appendix.

#### Research Question 1: How does social exclusion influence information use during risky decision-making?

A main effect of Cyberball Condition indicated that, contrary to **Hypothesis 1a**, adolescents made a greater number of risky decisions after social inclusion than after social exclusion (*Est*. = 0.11, *SE* = 0.03, *p* = < .001). Regarding information use in the CCT in general, results indicated that all adolescents reduced the number of risky choices when gain amounts were smaller (*Est*. = −0.08, *SE* = 0.01, *p* = <.001) and increased the number of risky choices when loss amounts were smaller (*Est*. = 0.11, *SE* = 0.01, *p* = <.001) and the probability of loss was smaller (*Est*. = 0.21, *SE* = 0.02, *p* = <.001). Results regarding the hypothesized decreases in the sensitivity to gain amounts, loss amounts, and loss probabilities with social exclusion (**Hypothesis 1b**) revealed significant interactions between Cyberball Condition and Gain Amount (*Est*. = 0.03, *SE* = 0.00, *p* = < .001), Cyberball Condition and Loss Amount (*Est*. = −0.04, *SE* = 0.00, *p* = <0.001), as well as Cyberball Condition and Loss Cards (*Est*. = −0.02, *SE* = 0.01, *p* = < 0.001). Contrary to the hypothesis, the pattern of results indicated that adolescents were more sensitive to differences in gain amounts, loss amounts, and loss probabilities when they were socially excluded than when they were included.

#### Research Question 2: Can the possibility to compare performance with the perpetrators of social exclusion bolster these effects?

Overall, there was no effect of Feedback Group on the number of risky decisions (*p* = 0.789) and no interaction between Feedback Group and Cyberball Condition (*p* = 0.440) that would indicate that social rank feedback increased effects of social exclusion (**Hypothesis 2a**). There was a small effect of social rank increasing the sensitivity to Loss Amount (*Est*. = 0.03, *SE* = 0.01, *p* = 0.050) and significant three-way interactions between Cyberball Condition, Feedback Group, and Loss Amount (*Est*. = 0.77, *SD* = 0.37, *p* = 0.036) as well as between Cyberball Condition, Feedback Group, and Gain Amount (*Est*. = 0.77, *SD* = 0.37, *p* = 0.036). Simple effects contrasting smaller and larger amounts of gain and loss suggested that adolescents were sensitive to amounts of loss and gain in all conditions except for the inclusion condition in the individual feedback group (see Table 5). The pattern of results indicated that, contrary to **Hypothesis 2b**, social rank increased sensitivity to gains and losses, particularly in the social inclusion condition.

#### Research Question 3: Are there developmental differences in combined effects of social exclusion and social rank feedback on information use during risky decision-making in adolescence?

**Fig. 5:**
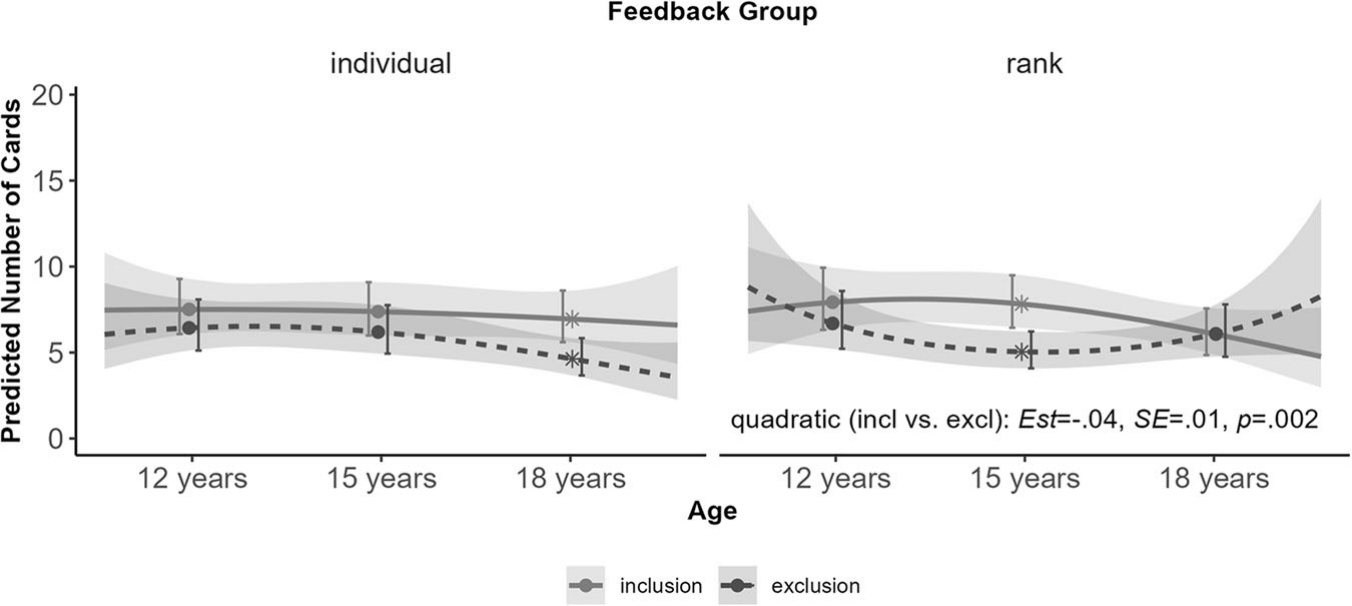
Developmental differences in the effect of Cyberball Condition on the number of cards turned over in the Columbia Card Task (CCT) as a function of Feedback Group (individual vs. social rank). Asterisks indicate that differences between social inclusion and social exclusion conditions were significant at *p* < 0.001

Regarding the hypotheses about developmental differences in the use of information in the CCT (**Hypothesis 3a**), significant interactions between linear Age and Gain Amount (*Est*. = −2.07, *SE* = 1.01, *p* = 0.041), linear Age and Loss Amount (*Est*. = 3.73, *SE* = 1.10, *p* = < 0.001), and linear Age and Loss Cards (*Est*. = 6.85, *SE* = 1.38, *p* = <0.001) suggested that adolescents became more sensitive to information about gain amounts, loss amounts, and loss probabilities with age. The pattern of results indicating whether the effects of social manipulation on risky decision-making are maximal in mid-adolescence (**Hypothesis 3b**), is shown in Fig. 5. As expected, the effect of social exclusion was modulated by quadratic Age and Feedback Group (*Est*. = 5.76, *SD* = 1.93, *p* = 0.003), indicating a significant peak in the effect of social exclusion in mid-adolescence for the group that received social rank feedback. Further examination by simple effects at the middle of the three age groups (12 years, 15 years, and 18 years of age) revealed that only late adolescents in the individual feedback group (*Est*. = 0.14, *SE* = 0.16, *p* = 0.389) and middle adolescents in the social rank group (*Est*. = 0.20, *SE* = 0.16, *p* = 0.201) significantly reduced risky decisions after social exclusion. There was no main effect for linear or quadratic Age (*p* = 0.081 and *p* = 0.919, respectively) and no further two-way interactions between Cyberball Condition, Feedback Group, and Age (all *p’s* ≥ 0.099).

**Fig. 7:**
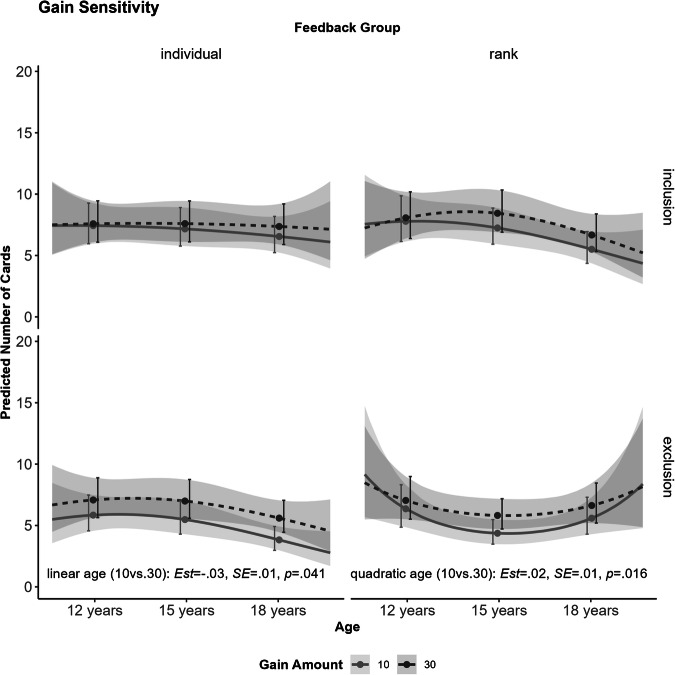
Developmental differences in the sensitivity to Gain Amounts (10 vs. 30 points) on the number of cards turned in the Columbia Card Task (CCT) as a function of Cyberball Condition (inclusion vs. exclusion) and Feedback Group (individual vs. social rank)

**Fig. 8:**
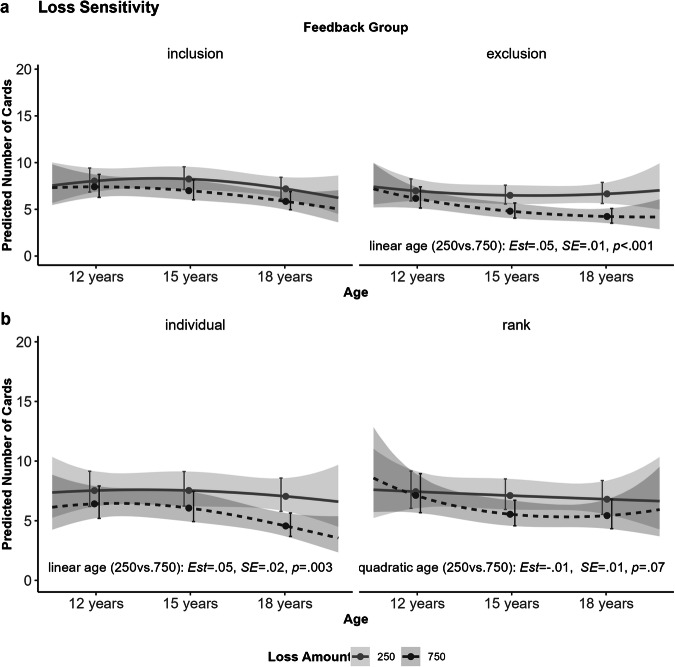
Developmental differences in the sensitivity to Loss Amounts (250 vs. 750 points) on the number of cards turned over in the Columbia Card Task (CCT) as a function of (**a**) Cyberball Condition (inclusion vs. exclusion) and (**b**) Feedback Group (individual vs. social rank)

**Fig. 9:**
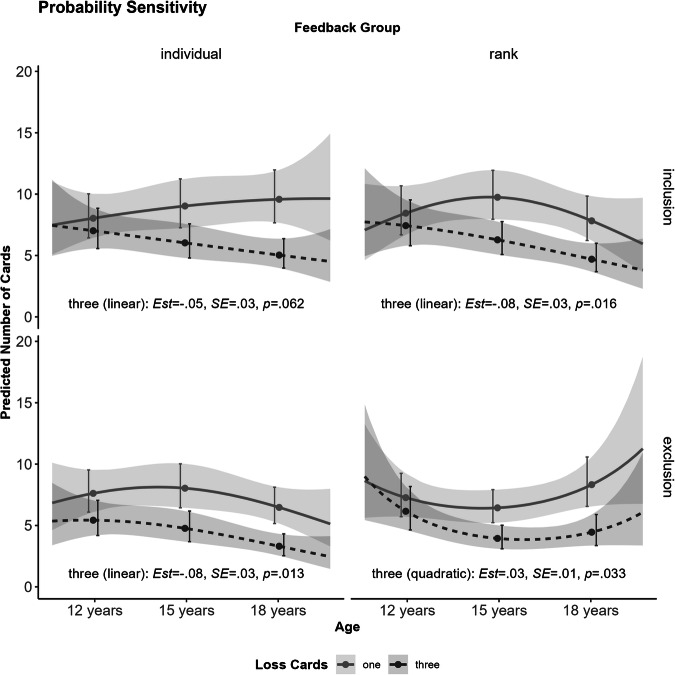
Developmental differences in the sensitivity to Loss Cards (one vs. three cards) on the number of cards turned over in the Columbia Card Task (CCT) as a function of Cyberball Condition (inclusion vs. exclusion) and Feedback Group (individual vs. social rank)

Higher order interactions between Cyberball Condition, Feedback Group, Age, and sensitivity to gain amounts and loss amounts (see Table 4) suggested that adolescents increasingly became more sensitive to differences in gain amounts and loss amounts with age in the exclusion condition of the individual feedback group, mirroring the greater reduction in the number of risky decisions after social exclusion in late adolescence. Thus, in the social rank feedback group, there was a peak in sensitivity to gain and loss amounts after social exclusion in mid-adolescence, again reflecting the greater reduction in risky decisions after social exclusion in the mid of the adolescent age range of this group (see Fig. 7 and Fig. 8). For the number of loss cards, results suggested a general increase in the sensitivity to loss probability with age, mostly induced by a decrease in risky decisions when loss probability was higher. In the inclusion condition of the individual feedback group, however, adolescents also increasingly engaged in risky decisions with age when loss probability was low (see Fig. 9). A more detailed report on these higher-order interactions can be found in Appendix B.

## Discussion

It has been proposed that mid-adolescents are particularly susceptible to social influences due to neurodevelopmental changes that increase their propensity to engage in risky behaviors when in the presence of their peers (Shulman et al., 2016). Although it has been postulated that this elevated propensity for risk-taking can be attributed to a fear of being excluded from the peer group (Tomova et al., 2021), the consequences of social ostracism on adolescent risk-taking behavior have thus far only been empirically examined in the context of simulated driving (Falk et al., 2014; Peake et al., 2013). Furthermore, it is believed that the reorientation towards peers as opposed to close family members also implies that adolescents begin to align their behavior with social goals in order to achieve higher social status among peers (Op de Macks et al., 2017). However, peer influences and social comparison effects have been examined independently of one another. Moreover, the developmental changes across adolescence, which could elucidate the increase in sensitivity to social manipulations and the mechanisms through which feelings of social exclusion and social comparison may elevate risk-taking tendencies in adolescence, remain insufficiently explored. The present study sought to investigate the impact of social exclusion and social comparison on risky decision-making across adolescence (11–19 years), with an additional focus on information processing related to gains, losses, and loss probabilities. Interestingly, the findings suggest that combined effects of social exclusion and social rank feedback can enhance adolescents’ consideration of information surrounding risk, decreasing tendencies to engage in risky decisions.

### How Does Social Exclusion Influence Information Use During Risky Decision-Making?

The analysis of emotional responses following social exclusion revealed that the manipulation effectively induced feelings of social exclusion. Participants reported receiving the ball significantly less frequently than expected during the exclusion scenario. Additionally, social exclusion notably reduced positive affect, mood, and feelings of belonging, aligning with findings from previous Cyberball studies (Hartgerink et al., 2015). However, contrary to expectations, social exclusion did not lead to an increase in risky decision-making. Conversely, adolescents demonstrated a reduction in risky decision-making as indexed by an increased awareness of the potential consequences of their actions. These findings challenge conventional assumptions linking social exclusion to elevated risk-taking behaviors during adolescence, as supported by previous research (Falk et al., 2014; Peake et al., 2013) and theoretical considerations (Blakemore, 2018; Tomova et al., 2021). Nevertheless, the study’s findings align with existing research indicating that other social influences, such as peer observation, can have both risk-inducing and risk-diminishing effects, depending on task-specific factors, contextual details, and age (e.g., Lorenz & Kray, 2022; Somerville et al., 2019).

For instance, empirical evidence indicates that adolescents, like adults and children, rely more on social information when faced with uncertainty (Ciranka et al., 2021a). Accordingly, peer influence can enhance confidence in decision-making, which may explain increased exploration under uncertainty (Lorenz & Kray, 2022; Romer et al., 2017). This is consistent with studies demonstrating increased risk-taking following social exclusion under uncertain conditions, such as simulated driving (Falk et al., 2014; Peake et al., 2013). In situations where risks and rewards are known, as in the CCT, peer influence may foster learning through social evaluation and valued contribution, highlighting information regarding which choices are more likely to result in rewards or losses (Ciranka et al., 2021b; Dahl et al., 2018; Silva et al., 2016). Accordingly, although all adolescents demonstrated a reduction in the number of risky decisions when gain amounts were smaller, loss amounts were larger, and loss probability was higher, it could be observed that social exclusion heightened sensitivity to such information in the CCT. Despite the prevailing view that adolescents are prone to impulsive and unconsidered actions, social influence may encompass social learning that can lead to adaptive behavior in both risk-taking and risk avoidance (Ciranka et al., 2021b; Dahl et al., 2018).

### Can the Possibility to Compare Performance with the Perpetrators of Social Exclusion Bolster These Effects?

The present study also illuminates the influence of social comparison on adolescent risk-taking behavior. Of particular interest is the observation that adolescents’ sensitivity to gain and loss amounts on risky decision-making varies based on the availability of social comparison feedback. Specifically, adolescents demonstrated heightened sensitivity to gains and losses following social exclusion, but also in the inclusion condition when social rank feedback was provided. In contrast, previous findings on social rank and disclosed feedback either showed no effect (Op de Macks et al., 2017) or indicated a strong motivation to gain social status despite potential losses (Cardoos et al., 2017). Therefore, social norms play a significant role in moderating the influence of social factors on adolescent risk-taking behavior. Research indicates that adolescents modify their risk propensity in accordance with the risk profile of their peer group (Cascio et al., 2015; Ciranka et al., 2021b; Tomova et al., 2021). This perspective aligns peer influence with a more reasoned form of risk-taking that involves a cost-benefit analysis, specifically considering the social benefits derived from risk-taking, such as gaining status and group belonging (Blakemore, 2018; Ciranka et al., 2021b; Tomova et al., 2021). In contrast to risk settings like the CCT, activities such as simulated driving often present opportunities to flout commonly understood traffic regulations, including basic rules like obeying traffic signals. Bidding scenarios, on the other hand, offer the possibility of gaining an advantage over others, which may result in social effects such as direct competition that might lead to a commitment to risk-taking irrespective of potential losses. These task characteristics provide avenues for self-presentation, fostering a perception of risk-taking as desirable or admired within social contexts. Consequently, in the context of the current task setting, it can be posited that cautious behavior may be perceived as a prevalent strategy among adolescents, as they endeavor to conform to peer expectations by achieving higher scores in the social rank feedback.

### Are There Developmental Differences in Combined Effects of Social Exclusion and Social Rank Feedback on Information Use During Risky Decision-Making in Adolescence?

Finally, the specific influence of social rank on risky decision-making became particularly evident in this study, due to the unique developmental differences on the influence of social rank on adolescent behavior. Although previous research has demonstrated that adolescents’ sensitivity to information surrounding risk increases with age (Figner et al., 2009), the current findings did not indicate a corresponding decrease in risky choices with age. A similar pattern of results was observed in previous studies that utilized the CCT in its version where feedback was not directly delivered (Figner et al., 2009; Somerville et al., 2019), suggesting that the CCT version employed in this study may promote the use of “cold” decision processes. However, an intriguing pattern emerged regarding developmental differences in the combined effects of social exclusion and social rank feedback. Specifically, middle adolescents demonstrated a more pronounced reduction in risky choices after social exclusion when comparing their performance with those who excluded them socially. That is, as suggested by theoretical accounts (Crone & Dahl, 2012; Shulman et al., 2016; Tomova et al., 2021) the peak of combined effects of social exclusion and social rank was in mid-adolescence. In contrast to the effects of peer observation, social exclusion did not result in an increase in risky choices in the CCT, as previously observed in younger age groups (aged 13–15 years, Somerville et al., 2019). Instead of acting impulsively, a more cautious approach was observed in mid-adolescence when feeling excluded, at least when performance was disclosed. This pattern of effects is comparable to the meta-analytic moderator effects found for the influence of peer observation on risky decision-making (Powers et al., 2022). There was overall no peak in the effect of peer observation in mid-adolescence but information about social norms, i.e., risk preferences displayed by peers, had a strong influence on whether peer observation had an influence on risky decisions, or not. Differences in the moderating effects of risk preference depending on the age range included in the analysis furthermore suggests developmental variations in moderating effects of social norms (Powers et al., 2022).

Accordingly, and in contrast to mid-adolescents, late adolescents exhibited reduced risky decision-making after social exclusion solely in the individual feedback group as opposed to the social rank feedback group, in this study. However, no significant age trend in the effect of social exclusion was found in the individual feedback group. Nevertheless, findings in the individual feedback group more closely resemble the findings on peer observation on risky decision-making in the CCT, where with increasing age more risks were taken when alone than when observed by peers (Somerville et al., 2019). It is noteworthy that social exclusion decreased feelings of control only in late adolescents in this study, suggesting qualitatively different effects of social exclusion between age groups. This finding also underscores the importance of considering multiple emotional facets beyond just valence effects in studies investigating social exclusion. Late adolescents may also place greater emphasis on non-social incentives than younger age groups, such as points as an index of their performance (for a review, see Kray et al., 2018). Therefore, social rank feedback may mitigate frustration resulting from frequent losses in the CCT by contextualizing low scores. This suggests that observing others attaining similarly low scores in the social rank feedback group may have reduced the inclination to become more cautious after social exclusion, as evidenced by the late adolescents in the individual feedback group.

Most intriguingly, the sensitivity to gain and loss amounts mirrored the developmental differences in the combined effects of social exclusion and social rank feedback on risky decisions. That is, the gain and loss sensitivity were maximal in late adolescence in the individual feedback group and during middle adolescence in the social rank feedback group. In contrast, the sensitivity to loss probability was rather insensitive to differences in feedback type. Overall, the sensitivity to loss probability increased with age, with a trend for adolescents to even increase risky decisions with age when loss probability was low in the individual feedback group. This suggests that, indeed, adolescents’ risk tendencies and changes therein are well reflected by sensitivity to information about known risks. In this regard, it is important to note that risk-taking is not inherently maladaptive; rather, it is a set of behaviors that foster exploration and independence, both of which are crucial for the achievement of developmental tasks during adolescence. As such, it may strongly depend on the context whether risk-taking and exploration or cautious behavior is advantageous. Many risky decision tasks in the laboratory actually encourage risk-taking and exploration, and more cautious behavior under peer influence may be disadvantageous in the long run, as in the Balloon Analog Risk Task (Lorenz & Kray, 2022). In other settings that allow for more learning, such as the Iowa Gambling Task (IGT), exploration is critical and has been found to increase with peer influence (Silva et al., 2016). In the case of the CCT, the study adds to the literature by showing that more cautious behavior after social exclusion is induced by greater adherence to available information. This highlights the benefits of using different task settings to examine the effects of social manipulation on risky decision making depending on the process of interest, which in this study was information use.

Taken together, the findings corroborate the hypothesis that mid-adolescents are particularly susceptible to social exclusion, particularly in settings comprising social comparison. This aligns with established views that emphasize adolescence as a sensitive developmental phase for social cognitive processing (Blakemore & Mills, 2014; Crone & Dahl, 2012). Interestingly, social exclusion did not lead to an increase in risky choices; instead, it was associated with a decrease in such behaviors. Moreover, the impact of social exclusion on risky decision-making was contingent upon the information available about the potential gains and losses involved. This suggests that feelings of exclusion and peer evaluation may promote cautious decision-making behaviors among adolescents, particularly in situations with low gains and high potential losses. In conclusion, this study contributes to the understanding of current theoretical frameworks that highlight the adaptive function of risk-taking among adolescents. In particular, it increases exploration and the acquisition of wisdom (Ciranka et al., 2021b; Romer et al., 2017), as well as the engagement in positive and prosocial risk behavior (Do et al., 2017; Duell & Steinberg, 2021; Kwon & Telzer, 2022). Considering factors such as peer influences, social comparison, and social norms, gives insights into the complexities of adolescent decision-making processes. Understanding these dynamics can inform interventions aimed at mitigating the negative effects of social exclusion and promoting healthier decision-making during adolescence. Specifically, the study suggests that peer contexts can alleviate motivation to find information that promote safe behavior when the outcomes promise peer group adherence.

### Limitations and Outlook

In discussing the present findings, it’s important to acknowledge several key points. Firstly, while the study covers a broad age range spanning early to late adolescence, which provides valuable insight into the developmental trajectory of social influences on risk-taking, the inclusion of even older age groups could offer additional insights. This is particularly relevant as emerging adults have been shown to engage in risky behaviors to a greater extent in real-life settings, and this study’s findings suggest a potential diminishing or reversal of social exclusion effects in older age groups. However, it’s worth noting that a study on emerging adults found no effects of social exclusion on risky decision-making in the CCT (Murphy, 2019). Additionally, longitudinal studies would provide the strongest evidence for understanding developmental changes over time.

Secondly, while the study contributes to theories and models regarding social influence in youth by considering the role of social exclusion and social comparison, it’s important to recognize that social exclusion may not be qualitatively or quantitatively distinct from other social influences on risky behavior, such as peer observation or direct influence. Future research should explore the comparative effects of different types of social influences or their interactions to determine the specific role of fear of social exclusion in social influences on risky decision-making during adolescence. Nevertheless, a significant strength of this study is the demonstration of differing effects of social exclusion depending on whether performance was disclosed and tied to social rank, or not, highlighting the importance of social standing in these effects. It will be of interest to observe how future studies address social rank when investigating peer influences, as there is considerable scope for further manipulation, including differences in rank position and risk-taking addressed to others versus the self (Do et al., 2017; Kwon & Telzer, 2022).

Thirdly, the study indicates that social exclusion affects the processing of information about risks and rewards, leading to increased sensitivity to such information. However, the specific cognitive processes involved remain unclear. The integration of electrophysiological measures, such as the electroencephalogram (EEG), could provide valuable insights in this regard. The inclusion of the CCT in the EEG would allow for the reflection on how sequential decision-making and feedback processing changes with development during adolescence. Especially, as the CCT is thought to offer good ecological validity, as it represents risky decisions that are more closely aligned with real-life experiences than other tasks. While there are numerous sequential risk-taking tasks, such as the Balloon Analog Risk Task or the Stoplight task, the CCT also allows for the simultaneous investigation of information use (Figner et al., 2009; Schonberg et al., 2011). This allows for the investigation of how adolescents and other age groups develop expectations about the occurrence of gains and losses in the CCT, which could be quantified with event-related potentials. Adolescents may develop fewer or less complex expectations than adults, reflecting their reduced information use and increased risk taking. In certain social interactions, however, this could be increased in adolescents, which would explain their being more flexible and socially adapted risk-takers than adults.

Lastly, it’s important to recognize that not all adolescents may exhibit cautious behavior following social exclusion. Individual differences, such as resistance to peer influence or rejection sensitivity, could account for the diverse findings ranging from increased to decreased risk-taking or no differences at all. While these influences were assessed in the current study, further investigation into individual differences will be explored in future publications dedicated to this specific aspect. Another crucial aspect not evaluated in this study is the sociocultural differences, primarily due to the predominance of White participants with German as at least one of their mother tongues.

## Conclusion

It has been assumed that adolescents will respond with risky decision-making when in fear of being excluded from their peer group. However, there is a paucity of research investigating the developmental differences that show this effect is maximal in adolescence, as well as the role of social comparison and underlying mechanisms. This study investigated how social exclusion affects risky decision-making across adolescence (11–19 years) and examined how factors like social comparison and information about gains, losses, and probabilities influence these effects. Contrary to initial expectations, social exclusion did not result in increased risky choices but rather in more cautious decision-making. The findings suggest that social exclusion can, under specific circumstances, positively influence risk adaptation by heightening sensitivity to relevant context information. Additionally, social exclusion and social rank had a differential impact on information usage, indicating that adolescence is a sensitive period for social cognition, which becomes increasingly complex with age. These findings corroborate recent accounts suggesting that peer influence can also have adaptive effects, fostering exploration and social learning.

## Figures and Tables

**Fig. 6:**
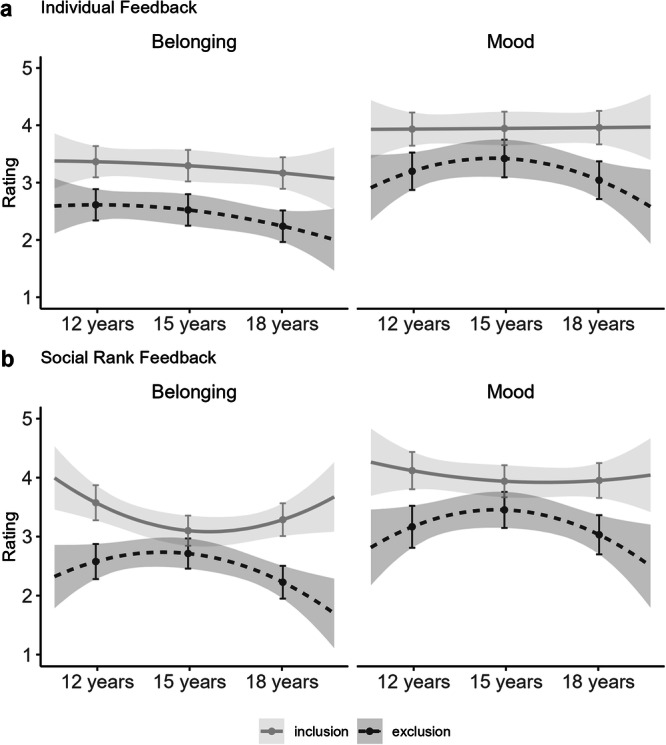
The effect of Cyberball Condition (Inlcusion/Exclusion) on the Mood and Belonging Scales of the Need Threat Questionnaire (NTQ) as a function of continuous Age in the (**a**) Individual Feedback group and the (**b**) Social Rank Feedback group

## Appendix

**Appendix A: Manipulation Check**

**SAM Scores**: This analysis focused on the three SAM subscales Affect, Arousal, and Control. The fixed factors of Age (continuous, linear and quadratic contrasts), Feedback Group (categorized as individual/social rank with −1/1 coding), and Timepoint were added to the model. The Timepoint factor contrasted emotional states at baseline with those observed following exposure to the social inclusion or exclusion conditions of the Cyberball paradigm. Furthermore, the Subscale factor, denoting Affect, Arousal, and Control dimensions, was treated as a within-subjects variable and sum-coded. The linear mixed effect model predicting SAM scores did not converge with the most complex random structure. Therefore, slopes for Cyberball Condition per participant were removed as they explained few variances. The final model included only a random slope for Subscale per participant. The results revealed highest order interactions between quadratic Age, Feedback Group, Timepoint, and Subscale (see Table 1). The pattern of results obtained through simple contrasts comparing SAM scores at baseline with scores after social inclusion and exclusion suggested that social exclusion significantly decreased positive affect in all groups (all *p*’s < 0.001) except for mid-adolescents in the individual feedback group (*p* = 0.216). Only early adolescents in the social rank feedback group reported increased positive affect after social inclusion compared to at baseline (*Est*. = 0.76, *SE* = 0.25, *p* = 0.004). Arousal was neither influenced by social inclusion (all *p*’s ≥ 0.387) nor by social exclusion (all *p*’s ≥ 0.244). Feelings of control were reduced by social exclusion only in late adolescents in both, the individual feedback group (*Est*. = −1.16, *SE* = 0.24, *p* < 0.001), and the social rank feedback group (*Est*. = −0.67, *SE* = 0.24, *p* = 0.010).

**Table 1:** Findings for the linear mixed effect model predicting SAM scores

*Predictors*	*Estimates*	95%*CI*.	*p*
(Intercept)	7.00	6.59–7.42	**<0.001**
Age [1: linear]	−2.70	−15.84 to 10.44	0.687
Age [2: quadratic]	3.71	−9.33 to 16.75	0.577
Feedback [individual vs social rank]	0.21	−0.36 to 0.79	0.469
Condition [1: baseline vs exclusion]	−1.31	−1.84 to −0.78	**<0.001**
Condition [2: baseline vs inclusion]	−0.15	−0.67 to 0.38	0.586
Subscale [1: Arousal versus Affect]	−2.21	−2.90 to −1.53	**<0.001**
Subscale [2: Affect versus Control]	−1.21	−1.85 to −0.58	**<0.001**
Age[1]:Feedback	5.93	−12.99 to 24.86	0.539
Age[2]:Feedback	−9.55	−28.58 to 9.48	0.325
Age[1]:Condition[1]	−7.87	−24.48 to 8.75	0.353
Age[2]:Condition[1]	−18.9	−35.39 to −2.41	**0.025**
Age[1]:Condition[2]	−3.93	−20.55 to 12.68	0.642
Age[2]:Condition[2]	1.66	−14.84 to 18.15	0.844
Feedback:Condition[1]	−0.54	−1.26 to 0.19	0.148
Feedback:Condition[2]	−0.04	−0.76 to 0.69	0.917
Age[1]:Subscale[1]	5.50	−16.19 to 27.19	0.619
Age[2]:Subscale[1]	−7.79	−29.32 to 13.74	0.478
Age[1]:Subscale[2]	20.30	0.32–40.27	**0.046**
Age[2]:Subscale[2]	−15.41	−35.24 to 4.42	0.128
Feedback:Subscale[1]	−0.12	−1.07 to 0.82	0.799
Feedback:Subscale[2]	0.02	−0.85 to 0.89	0.966
Condition[1]:Subscale[1]	1.26	0.51–2.00	**0.001**
Condition[2]:Subscale[1]	−0.42	−1.16 to 0.33	0.274
Condition[1]:Subscale[2]	0.38	−0.37 to 1.12	0.319
Condition[2]:Subscale[2]	−0.15	−0.89 to 0.60	0.701
Age[1]:Feedback:Condition[1]	10.89	−13.05 to 34.82	0.372
Age[2]:Feedback:Condition[1]	13.42	−10.65 to 37.49	0.274
Age[1]:Feedback:Condition[2]	−3.60	−27.53 to 20.33	0.768
Age[2]:Feedback:Condition[2]	3.27	−20.79 to 27.34	0.790
Age[1]:Feedback:Subscale[1]	−13.64	−44.88 to 17.61	0.392
Age[2]:Feedback:Subscale[1]	19.67	−11.74 to 51.09	0.219
Age[1]:Feedback:Subscale[2]	−24.4	−53.18 to 4.37	0.096
Age[2]:Feedback:Subscale[2]	11.9	−17.04 to 40.83	0.420
Age[1]:Condition[1]:Subscale[1]	8.13	−15.37 to 31.63	0.497
Age[2]:Condition[1]:Subscale[1]	24.72	1.40–48.05	**0.038**
Age[1]:Condition[2]:Subscale[1]	3.72	−19.78 to 27.22	0.756
Age[2]:Condition[2]:Subscale[1]	6.98	−16.35 to 30.31	0.557
Age[1]:Condition[1]:Subscale[2]	−23.12	−46.62 to 0.38	0.054
Age[2]:Condition[1]:Subscale[2]	17.5	−5.83 to 40.82	0.141
Age[1]:Condition[2]:Subscale[2]	−5.04	−28.54 to 18.46	0.674
Age[2]:Condition[2]:Subscale[2]	1.24	−22.09 to 24.56	0.917
Feedback:Condition[1]:Subscale[1]	0.40	−0.62 to 1.43	0.440
Feedback:Condition[2]:Subscale[1]	0.47	−0.55 to 1.50	0.366
Feedback:Condition[1]:Subscale[2]	0.78	−0.25 to 1.80	0.138
Feedback:Condition[2]:Subscale[2]	0.36	−0.67 to 1.38	0.496
Age[1]:Feedback:Condition[1]:Subscale[1]	−33.96	−67.80 to −0.11	**0.049**
Age[2]:Feedback:Condition[1]:Subscale[1]	−11.76	−45.80 to 22.28	0.498
Age[1]:Feedback:Condition[2]:Subscale[1]	−14.44	−48.29 to 19.40	0.403
Age[2]:Feedback:Condition[2]:Subscale[1]	−5.38	−39.41 to 28.66	0.757
Age[1]:Feedback:Condition[1]:Subscale[2]	13.02	−20.82 to 46.87	0.450
Age[2]:Feedback:Condition[1]:Subscale[2]	−9.02	−43.05 to 25.02	0.603
Age[1]:Feedback:Condition[2]:Subscale[2]	13.19	−20.65 to 47.04	0.445
Age[2]:Feedback:Condition[2]:Subscale[2]	9.37	−24.67 to 43.40	0.589
$${\bf{Random\; Effects}}$$			
Ω^2^	2.06		
Subject	2.90		
Subject by Subscale[1]	1.84		
Subject by Subscale[2]	1.40		
ICC	0.41		
N Subject	122		
Observations	1098		
Marginal R2 / Conditional R2	0.226/0.542		

Bold p-values indicate significant estimates at *p* ≤ .05.

**NTQ Scales (Mood and Belonging)**: For these scales, the contrast-coded fixed factor of Age (continuous, linear and quadratic contrasts) and Feedback Group (categorized as individual/social rank with −1/1 coding), as well as the factor Cyberball Condition (categorized as inclusion/exclusion with −1/1 coding) and the factor of Rating Type (categorized as mood/belonging with −1/1 coding) were incorporated. It was accounted for individual differences by treating participants as random effects and including a slope for both Subscale and Rating Type. The linear mixed effect model predicting the values of the NTQ subscales “Mood” and “Belonging” (see Table 2) revealed a higher order interaction between quadratic Age, Feedback Group, Cyberball Condition, and Rating Type. Simple effects of Cyberball Condition suggest that social exclusion decreased positive mood and feelings of belonging in both feedback groups and across age (see Fig. 6; all *Est*. = −0.39 to −1.05; all *p*’s = 0.022 – < 0.001).

**Table 2:** Findings for the linear mixed effect model predicting scores of the subscales Mood and Belonging of the Need Threat Questionnaire (NTQ)

*Predictors*	*Estimates*	95%*CI*.	*p*
(Intercept)	3.24	3.14–3.33	**<0.001**
Age [1: linear]	−1.98	−4.14 to 0.17	0.071
Age [2: quadratic]	−1.19	−3.34 to 0.97	0.280
Feedback [individual vs social rank]	−0.02	−0.12 to 0.08	0.691
Condition [inclusion vs exclusion]	−0.41	−0.48 to −0.33	**<0.001**
Rating Type [Mood vs Belonging]	−0.35	−0.39 to −0.31	**<0.001**
Age[1]:Feedback	0.27	−1.88 to 2.42	0.804
Age[2]:Feedback	−0.34	−2.50 to 1.81	0.755
Age[1]:Condition	−0.55	−2.19 to 1.09	0.510
Age[2]:Condition	−2.65	−4.29 to −1.00	**0.002**
Feedback:Condition	0.01	−0.06 to 0.09	0.727
Age[1]:Rating Type	−0.89	−1.84 to 0.06	0.067
Age[2]:Rating Type	0.82	−0.13 to 1.77	0.090
Feedback:Rating Type	−0.01	−0.05 to 0.04	0.794
Condition:Rating Type	−0.01	−0.05 to 0.02	0.344
Age[1]:Feedback:Condition	−0.34	−1.98 to 1.30	0.682
Age[2]:Feedback:Condition	1.31	−0.33 to 2.96	0.117
Age[1]:Feedback:Rating Type	−0.13	−1.08 to 0.82	0.786
Age[2]:Feedback:Rating Type	−0.19	−1.14 to 0.76	0.698
Age[1]:Condition:Rating Type	−0.1	−0.77 to 0.57	0.763
Age[2]:Condition:Rating Type	0.08	−0.59 to 0.75	0.811
Feedback:Condition:Rating Type	0.01	−0.03 to 0.03	0.788
Age[1]:Feedback:Condition:Rating Type	0.15	−0.52 to 0.82	0.664
Age[2]:Feedback:Condition:Rating Type	0.77	0.10–1.44	**0.025**
$${\bf{Random\; Effects}}$$			
Ω^2^	0.11		
Subject	0.27		
Subject by Condition	0.14		
Subject by Ratingtype	0.03		
ICC	0.79		
N Subject	120		
Observations	480		
Marginal R2/Conditional R2	0.366 / 0.870		

Bold p-values indicate significant estimates at *p* ≤ .05.

**Subjective Percentage of Balls Received in the Cyberball Paradigm:** To evaluate the subjective perception of ball receptions, the same fixed factors of Age Group, Feedback Group, and Cyberball Condition were utilized as in the previously described models. Including a unique slope per Cyberball Condition and participant resulted in convergence failure, why only participants were treated as a random effect without the consideration of individual slopes. The linear mixed effect model predicting the percentage of balls received in the Cyberball paradigm revealed a main effect of Cyberball Condition (see Table 3). Overall, adolescents recognized the exclusion from the ball tossing game by reporting a lower percentage of balls received in the exclusion than the inclusion condition (*Est*. = −6.77, *SE* = 0.51, *p* < 0.001). Estimated marginal means suggested that participants reported to have gained around 13.87% (*SE* = 1.42) of balls in the exclusion condition versus 27.03% (*SE* = 1.42) of balls in the inclusion condition. This means that participants slightly overrated the percentage of balls gained in the exclusion condition (5%) and underrated the percentage of balls gained in the inclusion condition ( ~33%). No other effects were found for the percentage of balls received in the Cyberball paradigm (all *p*’s ≥ 0.068).

**Table 3:** Findings for the linear mixed effect model predicting the subjective percentage of balls received in the Cyberball conditions

*Predictors*	*Estimates*	95%*CI*.	*p*
(Intercept)	18.84	17.26–20.41	< 0.001
Age [1: linear]	10.65	−13.83 to 35.14	0.392
Age [2: quadratic]	−22.35	−46.83 to 2.12	0.073
Feedback [individual vs social rank]	0.13	−1.44 to 1.71	0.870
Condition [inclusion vs exclusion]	−6.77	−7.78 to −5.75	<0.001
Age[1]:Feedback	8.40	−16.09 to 32.89	0.500
Age[2]:Feedback	4.16	−20.32to 28.63	0.738
Age[1]:Condition	−12.90	−28.64 to 2.85	0.108
Age[2]:Condition	−2.72	−18.42 to 12.98	0.733
Feedback:Condition	−0.25	−1.26 to 0.76	0.626
Age[1]:Feedback:Condition	−8.47	−24.21 to 7.27	0.290
Age[2]:Feedback:Condition	−3.98	−19.68 to 11.72	0.617
$${\bf{Random\; Effects}}$$			
Ω^2^	62.17		
Subject	44.49		
ICC	0.42		
N Subject	120		
Observations	239		
Marginal R2 / Conditional R2	0.320 / 0.603		

**Appendix B: Risky Decision-Making**

The full model including interaction terms as slopes per participant was too complex to converge as interaction terms explained very few variances. Therefore, a simplified random structure was used including independent random slopes for Exclusion Condition and the three information variables (Gain Amount, Loss Amount, and Loss Probability) per participant. The final model was: NumberCards ∼ Age ∗ Feedback Group ∗ Cyberball Condition ∗ (Gain Amount + Loss Amount + Loss Cards) + (1 + Cyberball Condition + Gain Amount + Loss Amount + Loss Cards | Subject).

**Details on Higher-Order Interactions**

For sensitivity to gain amounts, a three-way interaction between Cyberball Condition, Feedback Group, and Gain Amount (*Est*. = 0.02, *SD* = 0.00, *p* = <0.001), a four-way interaction between Cyberball Condition, Feedback Group, Gain Amount and linear Age (*Est*. = 0.77, *SD* = 0.37, *p* = 0.036), and a four-way interaction between Cyberball Condition, Feedback Group, Gain Amount and quadratic Age (*Est*. = 0.90, *SD* = 0.37, *p* = .014) revealed developmental differences in how combined effects of social exclusion and social rank influenced risky decisions. Simple contrasts revealed that in the individual feedback group sensitivity to gain amounts after social exclusion increased with age, while sensitivity to gain amounts was maximal in mid-adolescence after social exclusion in the social rank feedback group (see Fig. 7).

Concerning loss amounts, a similar pattern of results emerged but without the four-way interactions between Cyberball Condition, Feedback Group, Loss Amount and linear Age (*p* = 0.606) or quadratic Age (*p* = .863) becoming significant. Simple contrasts of a significant three-way interaction between Cyberball Condition, Loss Amount, and linear Age (*Est*. = −1.68, *SD* = 0.37, *p* = < 0.001) suggested an increase in the sensitivity to Loss Amount after social inclusion with age (see Fig. 8a). Moreover, simple contrasts of the significant three-way interaction between Feedback Group, Loss Amount, and quadratic Age (*Est*. = 2.43, *SD* = 1.11, *p* = .028) revealed a trend for a peak in loss amount sensitivity during mid-adolescence in the social rank but a linear increase with age in the individual feedback group (see Fig. 8b; Tables 4 and 5).

**Table 4:** Findings for the linear mixed effect model predicting the number of cards turned over in the Columbia Card Task (CCT) with Age as a continuous variable

*Predictors*	*Estimates*	95%*CI*.	*p*
(Intercept)	1.87	1.78–1.95	**<0.001**
Age [1: linear]	−6.32	−13.42 to 0.78	0.081
Age [2: quadratic]	−0.34	−6.98 to 6.29	0.919
Feedback [individual vs social rank]	−0.01	−0.10 to 0.07	0.789
Condition [inclusion vs exclusion]	0.11	0.06–0.16	**<0.001**
Gain Amount [10 points vs 30 points]	−0.08	−0.11 to −0.05	**<0.001**
Loss Amount [250 points vs 750 points]	0.11	0.08–0.14	**<0.001**
Loss Cards [1 card vs 3 cards]	0.21	0.17–0.24	**<0.001**
Age[1]:Feedback	−0.49	−7.58 to 6.61	0.893
Age[2]:Feedback	−3.31	−9.94 to 3.32	0.328
Age[1]:Condition	0.52	−3.24 to 4.29	0.785
Age[2]:Condition	−3.18	−6.96 to 0.60	0.099
Feedback:Condition	0.02	−0.03 to 0.07	0.440
Age[1]:Gain Amount	−2.07	−4.05 to −0.08	**0.041**
Age[2]:Gain Amount	0.88	−1.12–2.88	0.389
Age[1]:Loss Amount	3.73	1.58–5.88	**0.001**
Age[2]:Loss Amount	−0.47	−2.64 to 1.69	0.668
Age[1]:Loss Cards	6.85	4.14–9.56	**<0.001**
Age[2]:Loss Cards	−1.62	−4.33 to 1.10	0.244
Feedback:Gain Amount	−0.01	−0.03 to 0.02	0.693
Feedback:Loss Amount	0.03	0.00–0.06	**0.050**
Feedback:Loss Cards	0.02	−0.02–0.05	0.359
Condition:Gain Amount	0.03	0.02–0.04	**<0.001**
Condition:Loss Amount	−0.04	−0.05 to −0.03	**<0.001**
Condition:Loss Cards	−0.02	−0.03 to −0.01	**<0.001**
Age[1]:Feedback:Condition	3.68	−0.08 to 7.44	0.055
Age[2]:Feedback:Condition	5.76	1.98–9.54	**0.003**
Age[1]:Feedback:Gain Amount	−0.31	−2.29 to 1.68	0.760
Age[2]:Feedback:Gain Amount	−1.53	−3.53 to 0.47	0.135
Age[1]:Feedback:Loss Amount	0.84	−1.32 to 2.99	0.447
Age[2]:Feedback:Loss Amount	2.43	0.26–4.60	**0.028**
Age[1]:Feedback:Loss Cards	0.03	−2.68 to 2.74	0.981
Age[2]:Feedback:Loss Cards	1.14	−1.57 to 3.86	0.410
Age[1]:Condition:Gain Amount	−0.01	−0.73 to 0.72	0.988
Age[2]:Condition:Gain Amount	−0.50	−1.23 to 0.22	0.176
Age[1]:Condition:Loss Amount	−1.68	−2.41 to −0.95	**<0.001**
Age[2]:Condition:Loss Amount	−0.06	−0.78–0.67	0.882
Age[1]:Condition:Loss Cards	0.4	−0.35 to 1.14	0.294
Age[2]:Condition:Loss Cards	−0.15	−0.90 to 0.59	0.683
Feedback:Condition:Gain Amount	0.02	0.01–0.03	**<0.001**
Feedback:Condition:Loss Amount	−0.02	−0.03 to −0.01	**0.001**
Feedback:Condition:Loss Cards	−0.01	−0.02 to 0.00	0.254
Age[1]:Feedback:Condition:Gain Amount	0.77	0.05–1.50	**0.036**
Age[2]:Feedback:Condition:Gain Amount	0.90	0.18–1.63	**0.014**
Age[1]:Feedback:Condition:Loss Amount	−0.19	−0.92 to 0.54	0.606
Age[2]:Feedback:Condition:Loss Amount	−0.06	−0.79 to 0.67	0.863
Age[1]:Feedback:Condition:Loss Cards	1.04	0.30–1.79	**0.006**
Age[2]:Feedback:Condition:Loss Cards	0.18	−0.57 to 0.92	0.644
$${\bf{Random\; Effects}}$$			
Ω^2^	0.13		
Subject	0.22		
Condition by Subject	0.07		
Gain Amount by Subject	0.02		
Loss Amount by Subject	0.02		
Loss Cards by Subject	0.04		
ICC	0.74		
N Subject	122		
Observations	5856		
Marginal R2 / Conditional R2	0.185 / 0.787		

Bold p-values indicate significant estimates at *p* ≤ .05.

**Table 5:** Simple effects of gain and loss sensitivity as a function of Cyberball Condition and Feedback Group

Effect	Cyberball Condition	Feedback Group	*Estimate*	*SE*	*p*
${GainAmount}10-30$	inclusion	individual	−0.06	0.06	0.339
${GainAmount}10-30$	exclusion	individual	−0.24	0.06	**<0.001**
${GainAmount}10-30$	inclusion	rank	−0.15	0.06	**0.006**
${GainAmount}10-30$	exclusion	rank	−0.29	0.06	**<0.001**
${LossAmount}250-750$	inclusion	individual	0.12	0.07	0.075
${LossAmount}250-750$	exclusion	individual	0.32	0.07	**<0.001**
${LossAmount}250-750$	inclusion	rank	0.21	0.06	**<0.001**
${LossAmount}250-750$	exclusion	rank	0.29	0.06	**<0.001**

Bold p-values indicate significant estimates at *p* ≤ .05.

For the number of Loss Cards, there was a significant higher order interaction with Cyberball Condition, Feedback Group, and linear Age (*Est*. = 1.04, *SD* = 0.38, *p* = 0.006). Simple contrasts revealed linear increases in loss probability sensitivity across age in all groups and conditions. However, simple trends revealed that there were developmental trends in the number of risky decisions when loss probability was higher for all conditions except the inclusion condition of the individual feedback group (see Fig. 9). Developmental trends for risky decisions when loss probability was lower were all non-significant (all *p*’s ≥ 0.105).
